# 6-Formyl-2-meth­oxy-3-nitro­phenyl 4-toluene­sulfonate

**DOI:** 10.1107/S1600536808029711

**Published:** 2008-09-30

**Authors:** G. Ramachandran, R. Suresh, S. Sreedevi, Charles C Kanakam, V. Ramkumar

**Affiliations:** a Department of Chemistry, Valliammai Engineering College, SRM Nagar, Chennai, Tamil Nadu, India; b Department of Chemistry, Presidency College, Chennai, India; c Department of Chemistry, Indian Institute of Technology Madras, Chennai-36, Tamil Nadu, India

## Abstract

In the title compound, C_15_H_13_NO_7_S, the inter­planar angle between the two aromatic rings is 26.04 (3)°. The crystal structure is stabilized by C—H⋯O interactions.

## Related literature

For general background, see: Alford *et al.* (1991 ▶); Baldessarini (1987 ▶); Jiang *et al.* (1990 ▶); Spungin *et al.* (1992 ▶); Tharakan *et al.* (1992 ▶); Yachi *et al.* (1989 ▶). For related structures, see: Ramachandran *et al.* (2007 ▶); Ramachandran, Kanakam & Manivannan (2008 ▶); Ramachandran, Kanakam, Gunasekaran & Manivannan (2008 ▶); Ramachandran, Suresh, Chakkaravarthi *et al.* (2008 ▶); Manivannan *et al.* (2005*a* ▶,*b* ▶).
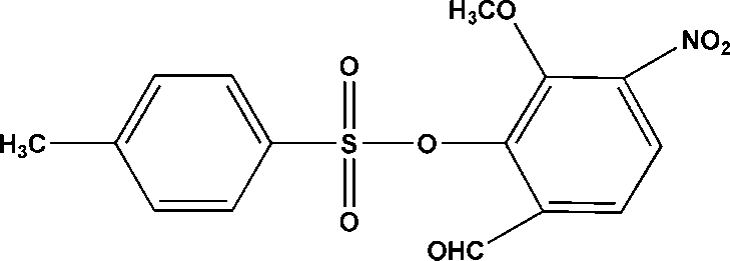


## Experimental

### 

#### Crystal data

C_15_H_13_NO_7_S
*M* *_r_* = 351.32
Triclinic, 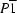

*a* = 8.1883 (16) Å
*b* = 9.5511 (19) Å
*c* = 10.530 (2) Å
α = 86.022 (3)°
β = 87.294 (3)°
γ = 73.588 (3)°
*V* = 787.8 (3) Å^3^
*Z* = 2
Mo *K*α radiation
μ = 0.24 mm^−1^
*T* = 298 (2) K
0.42 × 0.32 × 0.21 mm

#### Data collection

Bruker APEXII CCD area-detector diffractometer
Absorption correction: multi-scan (*SADABS*; Bruker, 1999 ▶) *T*_min_ = 0.905, *T*_max_ = 0.951
9176 measured reflections
3647 independent reflections
2897 reflections with *I* > 2σ(*I*)
*R*_int_ = 0.018

#### Refinement

*R*[*F* ^2^ > 2σ(*F* ^2^)] = 0.045
*wR*(*F* ^2^) = 0.129
*S* = 1.02
3647 reflections
223 parameters
H atoms treated by a mixture of independent and constrained refinement
Δρ_max_ = 0.25 e Å^−3^
Δρ_min_ = −0.24 e Å^−3^

### 

Data collection: *APEX2* (Bruker, 2004 ▶); cell refinement: *APEX2*; data reduction: *SAINT-Plus* (Bruker, 2004 ▶); program(s) used to solve structure: *SHELXS97* (Sheldrick, 2008 ▶); program(s) used to refine structure: *SHELXL97* (Sheldrick, 2008 ▶); molecular graphics: *ORTEP-3* (Farrugia, 1997 ▶); software used to prepare material for publication: *SHELXL97*.

## Supplementary Material

Crystal structure: contains datablocks global, I. DOI: 10.1107/S1600536808029711/bx2179sup1.cif

Structure factors: contains datablocks I. DOI: 10.1107/S1600536808029711/bx2179Isup2.hkl

Additional supplementary materials:  crystallographic information; 3D view; checkCIF report

## Figures and Tables

**Table 1:** Hydrogen-bond geometry (Å, °)

*D*—H⋯*A*	*D*—H	H⋯*A*	*D*⋯*A*	*D*—H⋯*A*
C2—H2⋯O6^i^	0.93	2.70	3.335 (3)	125

Symmetry code: (i) 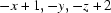.

**Fig. 1.**
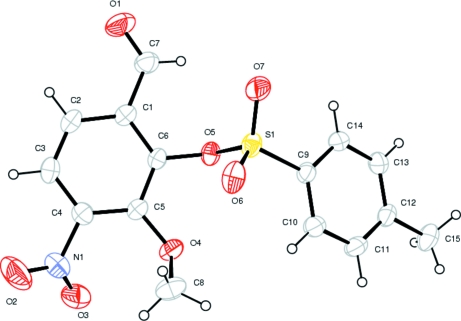
ORTEP of the molecule with atoms represented as 30% probability ellipsoids.

## supplementary crystallographic information

### Comment

Several compounds containing *p*-toluene sulfonate (PTS) moiety were used in the fields of biology and industry. The merging of lipids can be monitored using derivatives of *p*-toluene sulfonates (Yachi, *et al.*, 1989). This method has been used in studying the membrane fusion during the acrosome reaction (Spungin, *et al.*, 1992). PTS are used to purify human coagulation factor (Tharakan, *et al.*, 1992) in the study of viruses (Alford, *et al.*, 1991) and in the development of technology for linking photosensitizer to a model of monoclonal antibody (Jiang, *et al.*, 1990). In the field of pharmacology for the study of neuro pharmacology of s-adenosyl –L– methionine, PTS is used (Baldessarini, 1987). Because of the wide variety of biological importance of PTS, the synthesis of several substituted sulfonates and the study of their single-crystal XRD studies continues to be an interesting field of research. In the present paper, the synthesis and characterization by single-crystal study of the title compound is reported. In the title compound, the dihedral angle between the two aromatic rings is 26.04 (3)°.The geometric parameters agree with the reported values of similar structures (Manivannan *et al.*, 2005*a*, b; Ramachandran *et al.*, 2007). The angle between the O7—S1—O6 is 119.12 (11)°, which is greater than the tetrahedral angle, leading to the decrease in the O5—S1—C9 angle which is 97.9 (8)°. The *eclipsed* conformation of the sulfonyl moiety is confirmed by the torsion angle of O7—S1—C9—C14 = -19.3 (3)° and O6—S1—C9—C10 = 26.04 (19)°. The crystal packing is stabilized by Van der Waals interaction.

### Experimental

Acetylation of vanillin with acetic anhydride in presence of sodium acetate yielded acetyl vanillin. Powdered *o*-vanillin acetate was added to a stirred mixture of fuming HNO_3_ and concentrate H_2_SO_4_. The nitrated material was then hydrolyzed in 2% sodium hydroxide. The orange yellow solid was filtered and the filtrate was acidified to get the 4-nitro-2-hydroxy-3methoxy benzaldehyde. The benzaldehyde and triethylamine were dissolved in acetone and treated with 4-toluene sulfonyl chloride. The residue obtained was washed with 2% aqueous triethylamine solution to obtain the crude product. Diffraction quality crystals were obtained by recrystallizing the crude product from ethanol.

### Refinement

H atoms were positioned geometrically and refined using riding model with C—H = 0.93 Å and *U*_iso_(H) = 1.2*U*_eq_(C) for aromatic, C—H = 0.96 Å and *U*_iso_(H) = 1.5*U*_eq_(C) for CH_3_. The methyl groups were allowed to rotate but not to tip.

### Crystal data

**Table:**

C_15_H_13_NO_7_S	*Z* = 2
*M**_r_* = 351.32	*F*(000) = 364
Triclinic, *P*1	*D*_x_ = 1.481 Mg m^−^^3^
Hall symbol: -P 1	Mo *K*α radiation, λ = 0.71073 Å
*a* = 8.1883 (16) Å	Cell parameters from 873 reflections
*b* = 9.5511 (19) Å	θ = 2.6–26.3°
*c* = 10.530 (2) Å	µ = 0.24 mm^−^^1^
α = 86.022 (3)°	*T* = 298 K
β = 87.294 (3)°	Block, yellow
γ = 73.588 (3)°	0.42 × 0.32 × 0.21 mm
*V* = 787.8 (3) Å^3^	

### Data collection

**Table:**

Bruker APEXII CCD area-detector diffractometer	3647 independent reflections
Radiation source: fine-focus sealed tube	2897 reflections with *I* > 2σ(*I*)
graphite	*R*_int_ = 0.018
φ and ω scans	θ_max_ = 28.0°, θ_min_ = 1.9°
Absorption correction: multi-scan (SADABS; Bruker, 1999)	*h* = −10→10
*T*_min_ = 0.905, *T*_max_ = 0.951	*k* = −12→12
9176 measured reflections	*l* = −13→13

### Refinement

**Table:**

Refinement on *F*^2^	Primary atom site location: structure-invariant direct methods
Least-squares matrix: full	Secondary atom site location: difference Fourier map
*R*[*F*^2^ > 2σ(*F*^2^)] = 0.045	Hydrogen site location: inferred from neighbouring sites
*wR*(*F*^2^) = 0.129	H atoms treated by a mixture of independent and constrained refinement
*S* = 1.03	*w* = 1/[σ^2^(*F*_o_^2^) + (0.0677*P*)^2^ + 0.1932*P*] where *P* = (*F*_o_^2^ + 2*F*_c_^2^)/3
3647 reflections	(Δ/σ)_max_ = 0.001
223 parameters	Δρ_max_ = 0.25 e Å^−^^3^
0 restraints	Δρ_min_ = −0.24 e Å^−^^3^

### Special details

**Table:**

Geometry. All e.s.d.'s (except the e.s.d. in the dihedral angle between two l.s. planes) are estimated using the full covariance matrix. The cell e.s.d.'s are taken into account individually in the estimation of e.s.d.'s in distances, angles and torsion angles; correlations between e.s.d.'s in cell parameters are only used when they are defined by crystal symmetry. An approximate (isotropic) treatment of cell e.s.d.'s is used for estimating e.s.d.'s involving l.s. planes.
Refinement. Refinement of *F*^2^ against ALL reflections. The weighted *R*-factor *wR* and goodness of fit *S* are based on *F*^2^, conventional *R*-factors *R* are based on *F*, with *F* set to zero for negative *F*^2^. The threshold expression of *F*^2^ > σ(*F*^2^) is used only for calculating *R*-factors(gt) *etc*. and is not relevant to the choice of reflections for refinement. *R*-factors based on *F*^2^ are statistically about twice as large as those based on *F*, and *R*- factors based on ALL data will be even larger.

### Fractional atomic coordinates and isotropic or equivalent isotropic displacement parameters (Å^2^)

**Table:**

	*x*	*y*	*z*	*U*_iso_*/*U*_eq_	
C1	0.7558 (2)	−0.10359 (19)	0.96696 (18)	0.0478 (4)	
C2	0.6751 (2)	−0.1421 (2)	1.0781 (2)	0.0538 (5)	
H2	0.6431	−0.2284	1.0839	0.065*	
C3	0.6428 (2)	−0.0544 (2)	1.1783 (2)	0.0562 (5)	
H3	0.5877	−0.0801	1.2517	0.067*	
C4	0.6923 (2)	0.0729 (2)	1.17052 (18)	0.0517 (4)	
C5	0.7671 (2)	0.12023 (18)	1.06001 (18)	0.0456 (4)	
C6	0.7967 (2)	0.02845 (18)	0.95936 (17)	0.0426 (4)	
C7	0.7991 (3)	−0.2053 (2)	0.8626 (2)	0.0658 (6)	
C8	0.6935 (3)	0.3798 (2)	1.0658 (3)	0.0804 (7)	
H8A	0.5854	0.3754	1.0375	0.121*	
H8B	0.7267	0.4572	1.0182	0.121*	
H8C	0.6844	0.3981	1.1547	0.121*	
C9	0.9340 (2)	0.2467 (2)	0.67574 (17)	0.0476 (4)	
C10	0.9440 (3)	0.3730 (2)	0.7267 (2)	0.0585 (5)	
H10	0.8676	0.4155	0.7907	0.070*	
C11	1.0702 (3)	0.4352 (2)	0.6806 (2)	0.0613 (5)	
H11	1.0771	0.5211	0.7136	0.074*	
C12	1.1854 (3)	0.3732 (2)	0.58733 (19)	0.0541 (5)	
C13	1.1713 (3)	0.2464 (2)	0.5368 (2)	0.0603 (5)	
H13	1.2477	0.2039	0.4729	0.072*	
C14	1.0458 (3)	0.1830 (2)	0.58002 (19)	0.0569 (5)	
H14	1.0366	0.0987	0.5453	0.068*	
C15	1.3252 (3)	0.4399 (3)	0.5411 (3)	0.0757 (7)	
H15A	1.2901	0.5427	0.5541	0.114*	
H15B	1.3482	0.4254	0.4520	0.114*	
H15C	1.4263	0.3940	0.5876	0.114*	
N1	0.6741 (3)	0.1525 (2)	1.28715 (19)	0.0734 (5)	
S1	0.77841 (6)	0.16535 (5)	0.73509 (5)	0.05360 (17)	
O1	0.7587 (3)	−0.3163 (2)	0.8658 (2)	0.1075 (7)	
O2	0.5469 (3)	0.1609 (3)	1.3527 (2)	0.1279 (9)	
O3	0.7902 (3)	0.1989 (2)	1.31513 (18)	0.0950 (6)	
O4	0.81953 (17)	0.24287 (14)	1.04624 (14)	0.0588 (4)	
O5	0.88441 (15)	0.06651 (13)	0.85158 (12)	0.0497 (3)	
O6	0.63603 (17)	0.26914 (17)	0.78802 (15)	0.0685 (4)	
O7	0.7507 (2)	0.0679 (2)	0.64859 (16)	0.0786 (5)	
H7	0.862 (3)	−0.182 (3)	0.796 (2)	0.074 (7)*	

### Atomic displacement parameters (Å^2^)

**Table:**

	*U*^11^	*U*^22^	*U*^33^	*U*^12^	*U*^13^	*U*^23^
C1	0.0396 (8)	0.0408 (8)	0.0614 (11)	−0.0089 (7)	−0.0029 (8)	−0.0005 (8)
C2	0.0463 (10)	0.0454 (9)	0.0704 (12)	−0.0169 (8)	−0.0019 (9)	0.0074 (9)
C3	0.0443 (10)	0.0623 (11)	0.0601 (11)	−0.0158 (8)	0.0031 (8)	0.0110 (9)
C4	0.0417 (9)	0.0566 (10)	0.0536 (10)	−0.0085 (8)	0.0008 (8)	−0.0053 (8)
C5	0.0359 (8)	0.0404 (8)	0.0597 (10)	−0.0099 (7)	−0.0024 (7)	0.0002 (8)
C6	0.0331 (8)	0.0425 (8)	0.0507 (9)	−0.0098 (6)	−0.0006 (7)	0.0032 (7)
C7	0.0707 (14)	0.0507 (11)	0.0770 (15)	−0.0181 (10)	0.0055 (12)	−0.0123 (10)
C8	0.0891 (17)	0.0435 (11)	0.1050 (19)	−0.0107 (11)	−0.0060 (15)	−0.0103 (12)
C9	0.0500 (10)	0.0507 (9)	0.0465 (9)	−0.0226 (8)	−0.0023 (7)	0.0022 (7)
C10	0.0620 (12)	0.0551 (11)	0.0620 (12)	−0.0225 (9)	0.0110 (9)	−0.0115 (9)
C11	0.0699 (13)	0.0498 (10)	0.0722 (13)	−0.0295 (10)	0.0039 (10)	−0.0093 (9)
C12	0.0572 (11)	0.0548 (10)	0.0553 (10)	−0.0262 (9)	−0.0025 (9)	0.0082 (9)
C13	0.0651 (12)	0.0697 (12)	0.0516 (11)	−0.0289 (10)	0.0128 (9)	−0.0075 (9)
C14	0.0693 (12)	0.0589 (11)	0.0516 (10)	−0.0324 (10)	0.0041 (9)	−0.0100 (9)
C15	0.0691 (14)	0.0796 (15)	0.0879 (16)	−0.0416 (12)	0.0041 (12)	0.0126 (13)
N1	0.0743 (13)	0.0818 (13)	0.0614 (11)	−0.0165 (11)	0.0068 (10)	−0.0140 (10)
S1	0.0482 (3)	0.0630 (3)	0.0554 (3)	−0.0270 (2)	−0.0066 (2)	0.0082 (2)
O1	0.1421 (19)	0.0669 (11)	0.1292 (17)	−0.0530 (12)	0.0317 (14)	−0.0372 (11)
O2	0.1049 (16)	0.185 (2)	0.0988 (16)	−0.0444 (17)	0.0474 (14)	−0.0604 (17)
O3	0.1123 (15)	0.1068 (14)	0.0786 (12)	−0.0459 (13)	−0.0068 (11)	−0.0262 (11)
O4	0.0565 (8)	0.0453 (7)	0.0785 (9)	−0.0205 (6)	0.0023 (7)	−0.0070 (6)
O5	0.0402 (6)	0.0538 (7)	0.0535 (7)	−0.0130 (5)	0.0029 (5)	0.0052 (6)
O6	0.0433 (7)	0.0758 (9)	0.0808 (10)	−0.0136 (7)	−0.0020 (7)	0.0215 (8)
O7	0.0926 (12)	0.0985 (12)	0.0683 (9)	−0.0644 (10)	−0.0141 (8)	0.0003 (9)

### Geometric parameters (Å, °)

**Table:**

C1—C6	1.390 (2)	C9—C14	1.381 (3)
C1—C2	1.395 (3)	C9—S1	1.7417 (18)
C1—C7	1.481 (3)	C10—C11	1.385 (3)
C2—C3	1.362 (3)	C10—H10	0.9300
C2—H2	0.9300	C11—C12	1.372 (3)
C3—C4	1.382 (3)	C11—H11	0.9300
C3—H3	0.9300	C12—C13	1.391 (3)
C4—C5	1.397 (3)	C12—C15	1.507 (3)
C4—N1	1.469 (3)	C13—C14	1.378 (3)
C5—O4	1.353 (2)	C13—H13	0.9300
C5—C6	1.391 (3)	C14—H14	0.9300
C6—O5	1.396 (2)	C15—H15A	0.9600
C7—O1	1.195 (3)	C15—H15B	0.9600
C7—H7	0.90 (3)	C15—H15C	0.9600
C8—O4	1.439 (3)	N1—O2	1.208 (3)
C8—H8A	0.9600	N1—O3	1.212 (3)
C8—H8B	0.9600	S1—O7	1.4157 (17)
C8—H8C	0.9600	S1—O6	1.4194 (16)
C9—C10	1.378 (3)	S1—O5	1.6206 (13)
			
C6—C1—C2	118.45 (17)	C9—C10—H10	120.7
C6—C1—C7	122.12 (18)	C11—C10—H10	120.7
C2—C1—C7	119.42 (18)	C12—C11—C10	121.56 (18)
C3—C2—C1	120.55 (17)	C12—C11—H11	119.2
C3—C2—H2	119.7	C10—C11—H11	119.2
C1—C2—H2	119.7	C11—C12—C13	118.60 (17)
C2—C3—C4	119.79 (18)	C11—C12—C15	120.86 (19)
C2—C3—H3	120.1	C13—C12—C15	120.5 (2)
C4—C3—H3	120.1	C14—C13—C12	121.05 (19)
C3—C4—C5	122.26 (18)	C14—C13—H13	119.5
C3—C4—N1	117.05 (18)	C12—C13—H13	119.5
C5—C4—N1	120.54 (18)	C13—C14—C9	118.86 (18)
O4—C5—C6	118.41 (16)	C13—C14—H14	120.6
O4—C5—C4	125.31 (17)	C9—C14—H14	120.6
C6—C5—C4	116.20 (16)	C12—C15—H15A	109.5
C1—C6—C5	122.61 (16)	C12—C15—H15B	109.5
C1—C6—O5	119.59 (16)	H15A—C15—H15B	109.5
C5—C6—O5	117.56 (15)	C12—C15—H15C	109.5
O1—C7—C1	122.8 (2)	H15A—C15—H15C	109.5
O1—C7—H7	120.0 (16)	H15B—C15—H15C	109.5
C1—C7—H7	117.2 (16)	O2—N1—O3	124.0 (2)
O4—C8—H8A	109.5	O2—N1—C4	117.3 (2)
O4—C8—H8B	109.5	O3—N1—C4	118.5 (2)
H8A—C8—H8B	109.5	O7—S1—O6	119.12 (11)
O4—C8—H8C	109.5	O7—S1—O5	107.06 (9)
H8A—C8—H8C	109.5	O6—S1—O5	107.89 (8)
H8B—C8—H8C	109.5	O7—S1—C9	111.02 (10)
C10—C9—C14	121.31 (17)	O6—S1—C9	111.53 (9)
C10—C9—S1	119.04 (15)	O5—S1—C9	97.91 (8)
C14—C9—S1	119.64 (14)	C5—O4—C8	117.22 (16)
C9—C10—C11	118.60 (19)	C6—O5—S1	119.51 (10)
			
C6—C1—C2—C3	−2.3 (3)	C10—C11—C12—C15	−178.1 (2)
C7—C1—C2—C3	176.22 (18)	C11—C12—C13—C14	−0.7 (3)
C1—C2—C3—C4	−0.8 (3)	C15—C12—C13—C14	178.8 (2)
C2—C3—C4—C5	3.5 (3)	C12—C13—C14—C9	−0.5 (3)
C2—C3—C4—N1	−172.22 (18)	C10—C9—C14—C13	1.1 (3)
C3—C4—C5—O4	−179.50 (17)	S1—C9—C14—C13	−178.09 (17)
N1—C4—C5—O4	−3.9 (3)	C3—C4—N1—O2	−40.7 (3)
C3—C4—C5—C6	−2.9 (3)	C5—C4—N1—O2	143.5 (2)
N1—C4—C5—C6	172.72 (16)	C3—C4—N1—O3	135.6 (2)
C2—C1—C6—C5	2.9 (3)	C5—C4—N1—O3	−40.2 (3)
C7—C1—C6—C5	−175.55 (17)	C10—C9—S1—O7	161.44 (16)
C2—C1—C6—O5	177.10 (15)	C14—C9—S1—O7	−19.3 (2)
C7—C1—C6—O5	−1.3 (3)	C10—C9—S1—O6	26.04 (19)
O4—C5—C6—C1	176.49 (15)	C14—C9—S1—O6	−154.70 (16)
C4—C5—C6—C1	−0.4 (2)	C10—C9—S1—O5	−86.80 (17)
O4—C5—C6—O5	2.2 (2)	C14—C9—S1—O5	92.45 (17)
C4—C5—C6—O5	−174.69 (14)	C6—C5—O4—C8	124.5 (2)
C6—C1—C7—O1	−176.9 (2)	C4—C5—O4—C8	−58.9 (3)
C2—C1—C7—O1	4.7 (3)	C1—C6—O5—S1	93.43 (17)
C14—C9—C10—C11	−0.5 (3)	C5—C6—O5—S1	−92.07 (17)
S1—C9—C10—C11	178.74 (17)	O7—S1—O5—C6	−92.32 (14)
C9—C10—C11—C12	−0.8 (3)	O6—S1—O5—C6	37.02 (15)
C10—C11—C12—C13	1.4 (3)	C9—S1—O5—C6	152.75 (13)

### Hydrogen-bond geometry (Å, °)

**Table:**

*D*—H···*A*	*D*—H	H···*A*	*D*···*A*	*D*—H···*A*
C2—H2···O6^i^	0.93	2.70	3.335 (3)	125

Symmetry codes: (i) −*x*+1, −*y*, −*z*+2.
